# Supplementary material for: Genomic epidemiology of syphilis reveals independent emergence of macrolide resistance across multiple circulating lineages
Source: Nat Commun. 2019 Jul 22;10:3255. doi: 10.1038/s41467-019-11216-7 (PMC6646400; doi:10.1038/s41467-019-11216-7)
Supplement: Supplementary file 1 — Supplementary Information [file 41467_2019_11216_MOESM1_ESM.pdf]

Genomic epidemiology of syphilis reveals independent emergence of macrolide resistance across multiple circulating lineages

Beale et al.

Supplementary Information

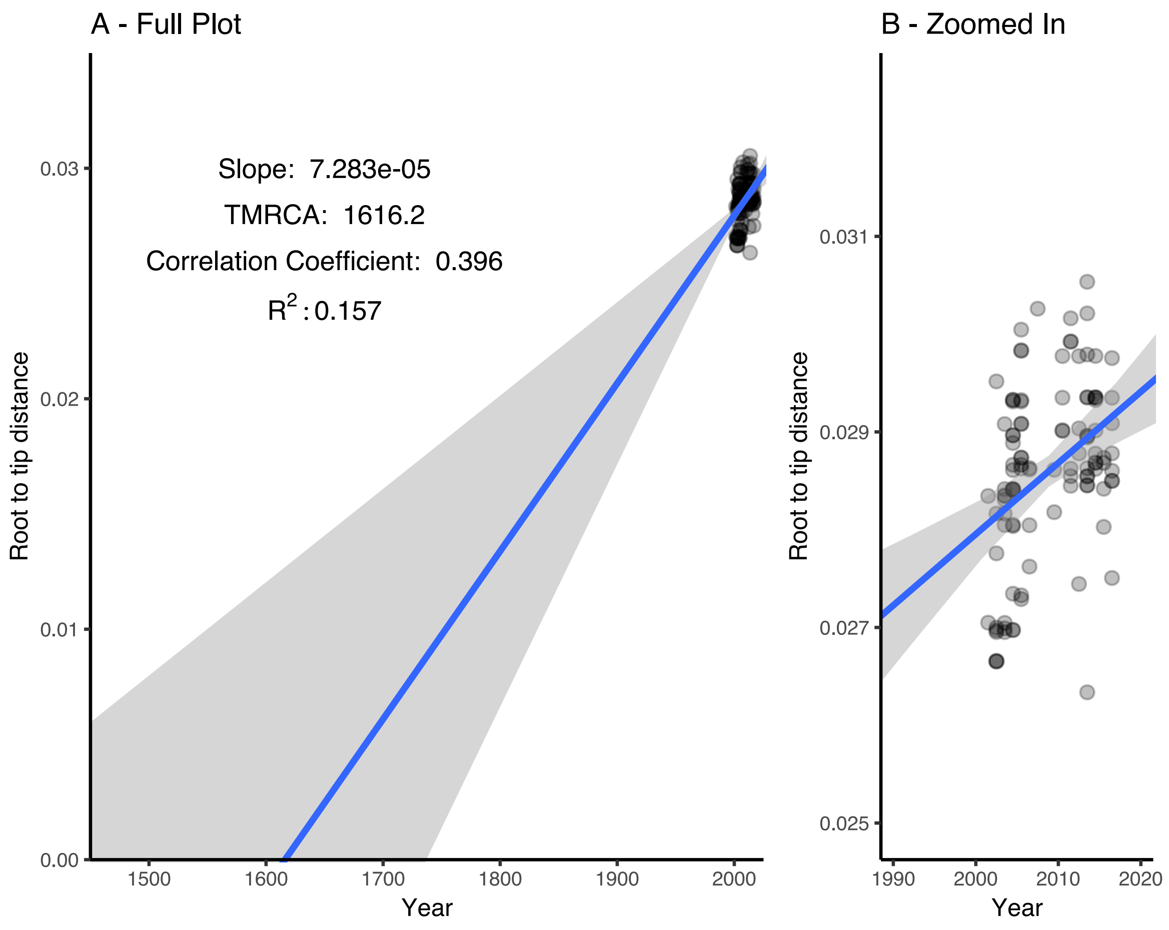

Supplementary Figure 1. Root-to-tip regression analysis of tip dates against branch lengths showing a correlation of 0.40 and R2 of 0.16, providing evidence for temporal signal in the Maximum Likelihood tree. Analysis performed in TempEst using clinically derived genomes from both Nichols and SS14 lineages. Plots show tip points and linear regression line (with standard error) for full timeline (A) and zoomed in to only include sampled tip dates (B). Each data point is coloured grey, with darker colours indicating multiple overlapping points.

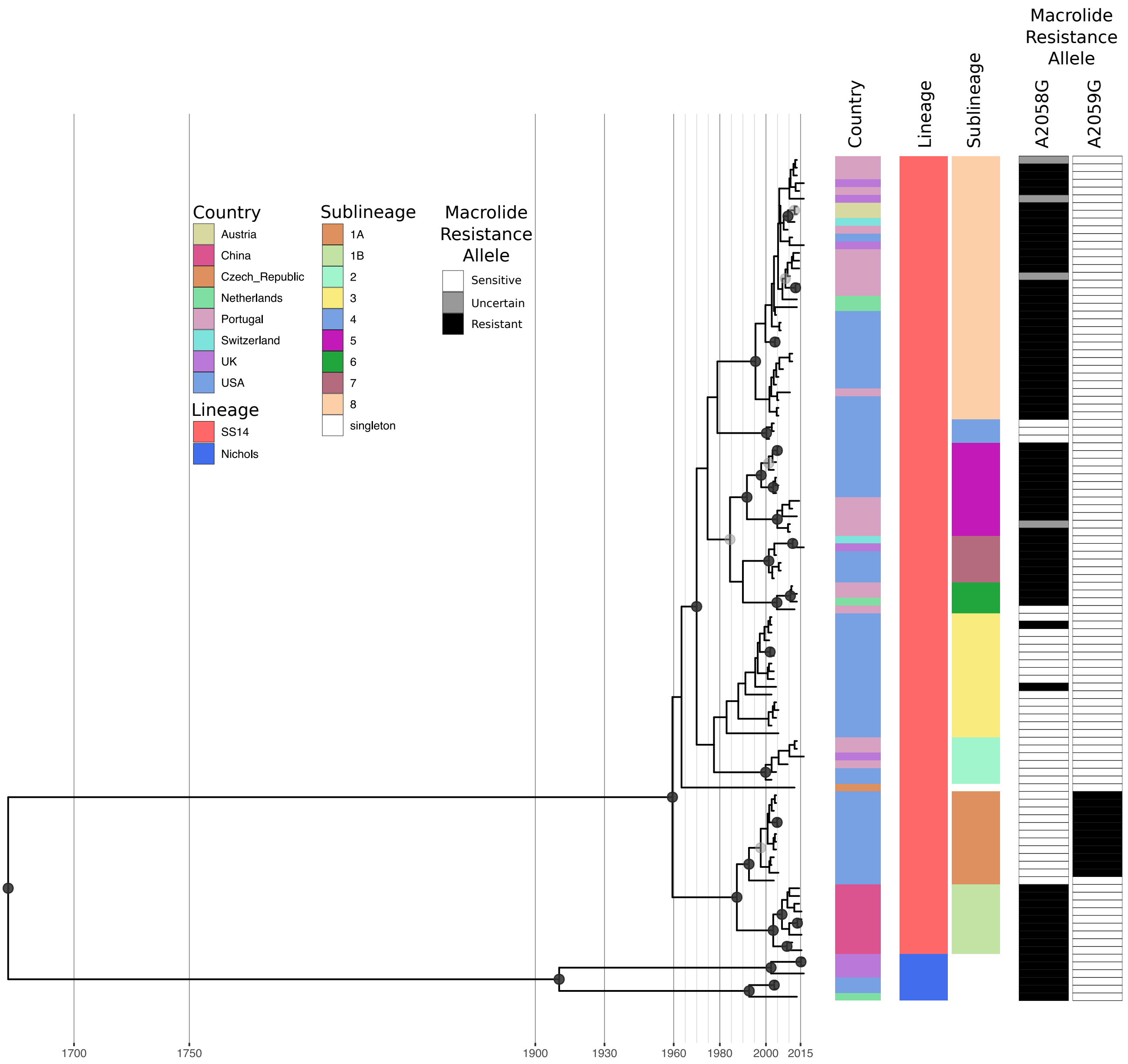

Supplementary Figure 2. Bayesian maximum credibility phylogeny expansion of discrete sub-lineages within SS14-lineage, with independent evolution of macrolide resistance. Time-scaled phylogeny of all recently clinically derived genomes. Coloured tracks indicate country of origin, lineage, sub-lineage, and presence of macrolide resistance conferring 23S rRNA SNPs (black=present, white=absent, grey=uncertain). Node points are shaded according to posterior support (black ≥96%, dark grey >91%, light grey >80%).

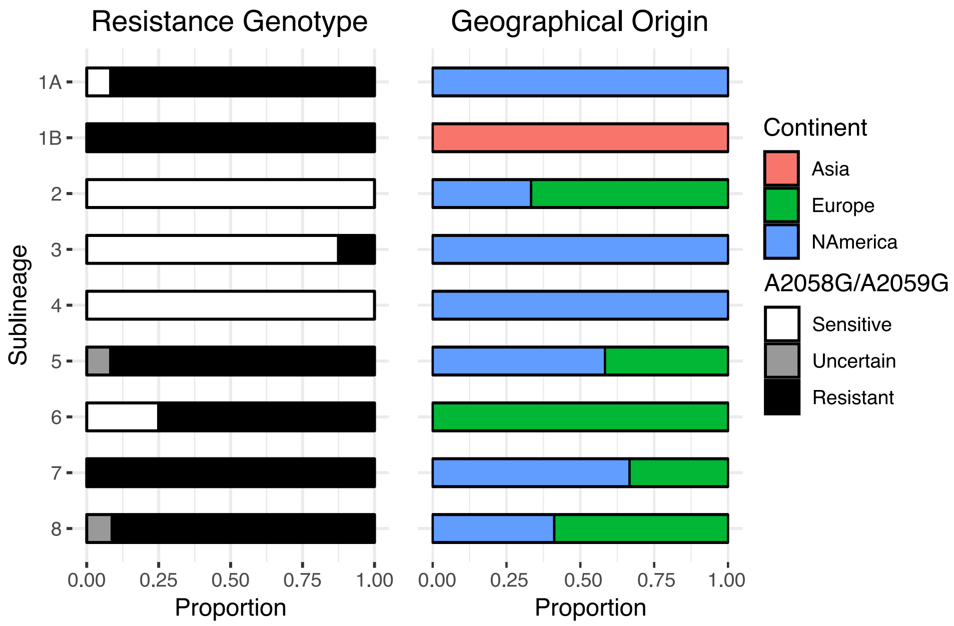

Supplementary Figure 3. Distribution of macrolide resistance genotype and geographical origin according to SS14 sub-lineage of recently clinically derived genomes. Four sub-lineages (one comprising sensitive samples, three comprising predominantly resistant samples) are geospatially admixed, containing sequences from both North America and Europe. Three other sub-lineages are composed entirely of samples from Seattle (USA, North America) including two predominantly macrolide sensitive lineages and one genotypically resistant lineage, as is one resistant lineage from China (Asia). Resistance genotypes: Resistant (Black), Sensitive (White), Uncertain (Grey). Continent of sampling: Asia (Pink), Europe (Green), North America (Blue).

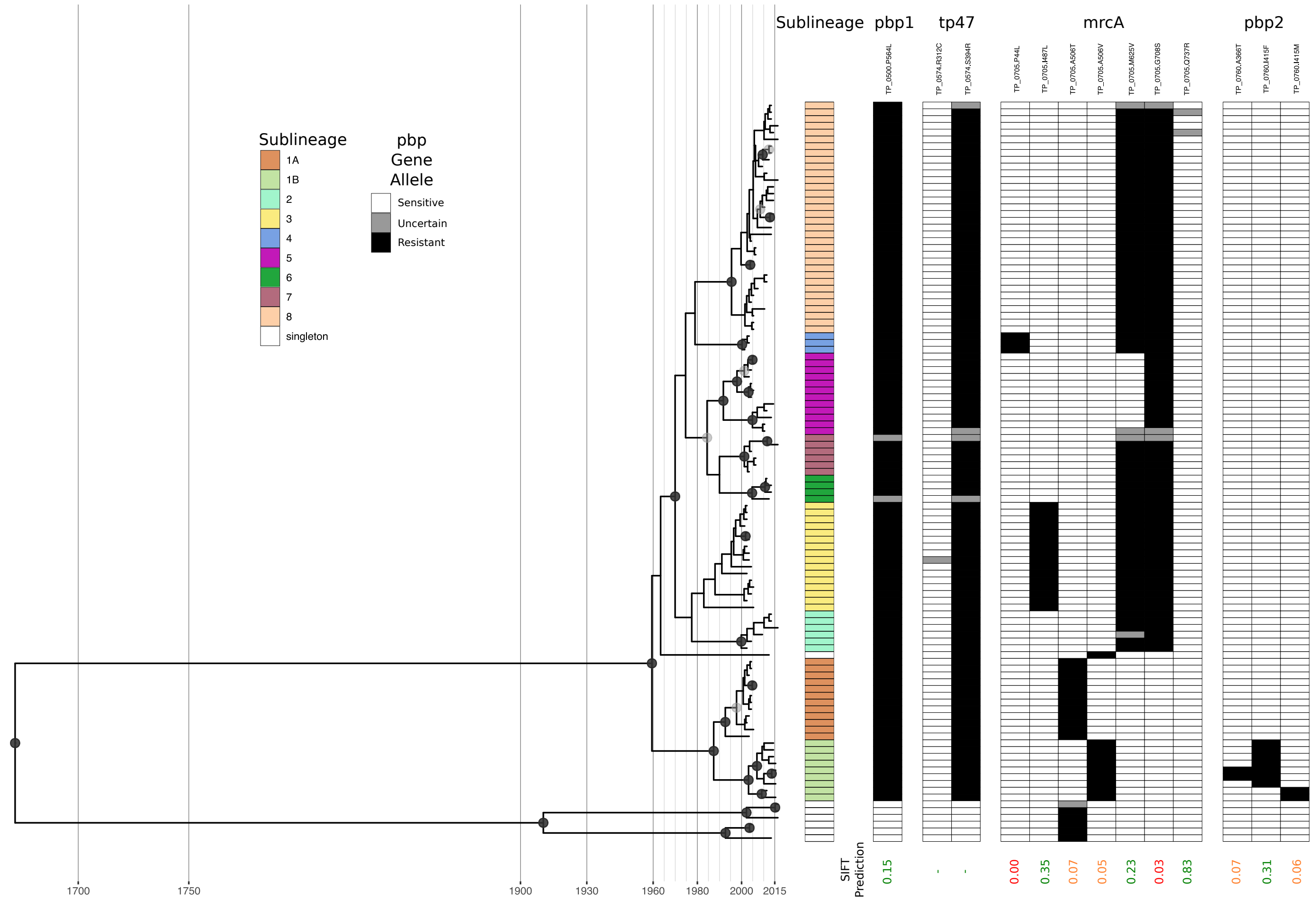

Supplementary Figure 4. Bayesian maximum credibility phylogeny showing non-synonymous SNPs predicted in penicillin binding protein genes. Tree contains only recently clinically derived genomes, and shows variants in *pbp1* (TPANIC_0500), *pbp2* (TPANIC_0760), *mrcA* (TPANIC_0705) and *Tp47* (TPANIC_0574). SNPs are relative to the Nichols reference sequence (NC_021490.2), and coloured tracks indicate sublineage and genotype of non-synonymous sites (black=present, white=absent, grey=uncertain). SIFT predictions of functional impact are shown for all genes except *Tp47*, where scores below

0.05 (coloured red) indicate a likely functional impact of the amino acid change; variants above this threshold but below 0.1 are indicated in orange, and variants above 0.1 are in green. Many SNPs are conserved by lineage, with some isolated *de novo* mutation. Within *mrcA*, amino acid position A506 is targeted by three independent non-synonymous SNPs.

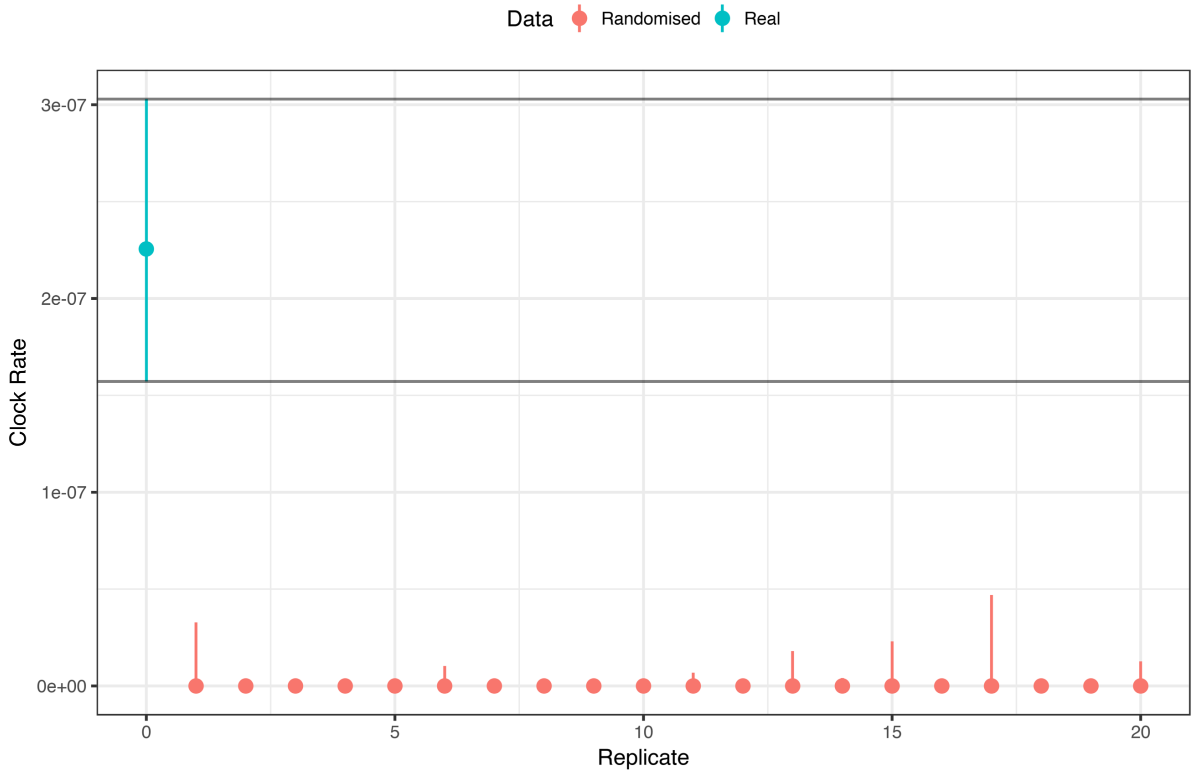

Supplementary Figure 5. Tip date resampling analysis shows temporal signal was not obtained by chance. Resampling performed using twenty datasets with randomised tip dates generated from the original Strict Clock analysis and run in BEAST under the same conditions. Median clock rate for the real tree was 2.26×10^{-7}, whilst all randomly assigned datasets gave substantially lower clock rates, with the highest median clock rate obtained at 1.90×10^{-12}. This indicates that the temporal signal observed in our tree was not obtained by chance, and provides further evidence for a temporal signal in the multiple sequence alignment. Real sample (blue), tip randomised samples (pink).
