# Supplementary material for: Genomic epidemiology of syphilis reveals independent emergence of macrolide resistance across multiple circulating lineages
Source: Nat Commun. 2019 Jul 22;10:3255. doi: 10.1038/s41467-019-11216-7 (PMC6646400; doi:10.1038/s41467-019-11216-7)
Supplement: Supplementary file 3 — Reporting Summary [file 41467_2019_11216_MOESM3_ESM.pdf]

natureresearch

Corresponding author(s): Mathew A Beale, Nicholas R Thomson

Last updated by author(s): May 7, 2019

Reporting Summary

Nature Research wishes to improve the reproducibility of the work that we publish. This form provides structure for consistency and transparency in reporting. For further information on Nature Research policies, see Authors & Referees and the Editorial Policy Checklist .

Statistics

For all statistical analyses, confirm that the following items are present in the figure legend, table legend, main text, or Methods section.

n/a	Confirmed	
☐	☒	The exact sample size (n) for each experimental group/condition, given as a discrete number and unit of measurement
☐	☒	A statement on whether measurements were taken from distinct samples or whether the same sample was measured repeatedly
☐	☒	The statistical test(s) used AND whether they are one- or two-sided<br>*Only common tests should be described solely by name; describe more complex techniques in the Methods section.*
☐	☒	A description of all covariates tested
☐	☒	A description of any assumptions or corrections, such as tests of normality and adjustment for multiple comparisons
☐	☒	A full description of the statistical parameters including central tendency (e.g. means) or other basic estimates (e.g. regression coefficient) AND variation (e.g. standard deviation) or associated estimates of uncertainty (e.g. confidence intervals)
☒	☐	For null hypothesis testing, the test statistic (e.g. F, t, r) with confidence intervals, effect sizes, degrees of freedom and P value noted<br>*Give P values as exact values whenever suitable.*
☐	☒	For Bayesian analysis, information on the choice of priors and Markov chain Monte Carlo settings
☒	☐	For hierarchical and complex designs, identification of the appropriate level for tests and full reporting of outcomes
☒	☐	Estimates of effect sizes (e.g. Cohen's d, Pearson's r), indicating how they were calculated

Our web collection on statistics for biologists contains articles on many of the points above.

Software and code

Policy information about availability of computer code

Data collection: No software was used

Data analysis: All data were analysed in R v3.5.1. Code used to generate Figures 1, 2 and 3, Supplementary Figures 2 - 4, as well as all statistics used in the primary manuscript is available at doi:10.6084/m9.figshare.7688093. Figures 1 and 2 were generated in R v3.5.1 using ggplot2 v3.1.0 and ggtree v1.14.6. Figure 3 was generated using ggtree, ggplot2, phangorn 2.4.0, phytools v0.6-60 and dplyr v0.8.0.1, with further data reshaping using ape v5.2, reshape2 v1.4.3 and gridExtra v2.3.

Treponemal sequencing reads were prefiltered using a Kraken v0.10.6 database containing all bacterial and archaeal nucleotide sequences in RefSeq, plus mouse and human. Sequencing reads were trimmed for quality and adaptors using Trimmomatic v0.33. Readset binning and subsampling was performed using seqtk v1.0-r31 (https://github.com/lh3/seqtk). Where sequencing reads were unavailable for published genomes, perfect simulated reads were generated from assemblies using Fastaq v3.17.0 (https://github.com/sanger-pathogens/Fastaq).

Reference Sequence NC_021508.1 (SS14_v2) was masked for known recombinant, hypervariable and repetitive genes (positions described in Supplementary 2) using bedtools v2.17.0 'maskfasta' before mapping. Reads were mapped to the reference using BWA mem v0.7.17, followed by indel realignment using GATK v3.4-46, and duplicate marking using Picard-tools MarkDuplicates v1.127 (http://broadinstitute.github.io/picard/). Variant calling and consensus pseudosequences were generated using samtools v1.2 and bcftools v1.2.

Multiple sequence alignments were screened for evidence of recombination using Gubbins v1.4.10. Recombination-masked SNP-only alignments from Gubbins were used in IQ-Tree v1.6.3.

Joint ancestral reconstruction of SNPs on the maximum likelihood phylogeny was conducted using pyjar (https://github.com/simonrharris/pyjar). The output from pyjar was then used as input to rPincone (https://github.com/alexwailan/rpinecone), using a clustering threshold of 10 SNPs and a 'relateability threshold' of 3.

IQ-Tree ML phylogenies were analysed for evidence of temporal signal using TempEst v1.5, and raw branch lengths and dates were extracted from TempEst and used to plot Supplementary Figure 1 using ggplot2 v3.1.0 in R v3.5.1. Recombination-masked whole genome sequence alignments from Gubbins were used to determine the number of constant sites, and the alignments were then filtered to only include parsimony informative sites using Biopython v1.68. BEAST v1.8.2 was run on filtered alignments. Tip date resampling and randomised datasets were generated using the TipDatingBeast v1.0-8 package in R. Randomised BEAST runs were collated after running using TipDatingBeast, and then plotted using ggplot2 as Supplementary Figure 2.

Macrolide resistance alleles were inferred using ARIBA v2.12.1 against a database generated from reference sequence NR_076156.1. Penicillin binding protein variants were inferred using using ARIBA v2.12.1 against a database generated from gene sequences extracted from reference sequence NC_021490.2.

For additional reanalysis of competitively mapped reads, variant calling was performed on mapped and filtered reads using bcftools, then processed using 'bcf-to-minorvars_v1.2.py' (available at https://github.com/matbeale/Global_Syphilis_Phylo_2019), before collating the results of all samples using 'Collate_Minor_var_scans_v0.11.py' (available at https://github.com/matbeale/Global_Syphilis_Phylo_2019).

For manuscripts utilizing custom algorithms or software that are central to the research but not yet described in published literature, software must be made available to editors/reviewers. We strongly encourage code deposition in a community repository (e.g. GitHub). See the Nature Research guidelines for submitting code & software for further information.

Data

Policy information about availability of data

All manuscripts must include a data availability statement. This statement should provide the following information, where applicable:

- Accession codes, unique identifiers, or web links for publicly available datasets
- A list of figures that have associated raw data
- A description of any restrictions on data availability

Raw sequencing reads for all novel sequences were deposited at the European Nucleotide Archive (ENA) under project PRJEB20795. All accessions (both novel and previously published) used in this project are listed in Supplementary Table 1, along with all metadata used for analysis in Figures 1, 2 and 3, and Supplementary Figures 1, 2, 3, 4 and 5.

Field-specific reporting

Please select the one below that is the best fit for your research. If you are not sure, read the appropriate sections before making your selection.

☒ Life sciences ☐ Behavioural & social sciences ☐ Ecological, evolutionary & environmental sciences

For a reference copy of the document with all sections, see nature.com/documents/nr-reporting-summary-flat.pdf

Life sciences study design

All studies must disclose on these points even when the disclosure is negative.

Sample size

Not applicable. This was an exploratory study to describe population structure in a field where little data is available. Eight UK samples were collected prospectively from clinical patients in 2016 as part of pilot work to establish the sequencing method directly from clinical samples. Sixty US samples were collected as part of a study of cerebrospinal fluid samples abnormalities conducted between 2001 and 2011 (a mixture of 21 patients with evidence of CSF infection and 38 patients without), and were sequenced retrospectively using residual genomic DNA. We also included 49 whole genomes published elsewhere, giving a total sample size of 122 samples. Of these, we could establish that 109 were recently derived from clinical patients, with the remainder potentially subject to multiple passages in the rabbit model.

Data exclusions

Additional global sequences were considered for the study but were excluded due to duplication (multiple instances of the same genome sequenced), low sequencing coverage (insufficient for accurate phylogenetic inference or macrolide SNP allele calling), or substantial contamination even after extensive data cleaning and filtering using the pipeline described. Of the 122 sequences, 13 were either described as heavily passaged in the literature, or there was limited information available describing provenance - these samples were excluded from temporal analysis as noted in the text.

Replication

This was a sequencing study using a unique sample collection. Replication of the sample set is not possible at this time. The overall population structure described broadly replicates that published by Arora et al, and phylogenetic analysis was separately reproduced using IQ-Tree and BEAST (in paper). We sequenced five genomes previously published elsewhere (described in results) - a phylogenetic analysis containing all samples in the study, as well as both versions of each genome demonstrated equivalent phylogenetic placement for these replicates (phylogeny not included). Macrolide resistance alleles were inferred from both the subsampled reads and the full readset - SNP calls were equivalent for each readset. We also recalled all variants using an alternative stringent competitive mapping approach. Apart from some minor discrepancies (which have been marked as uncertain in the manuscript) all sites were in agreement between the different methods.

All computer code used in the analyses and in the production of Figures 1-3 and Supplementary Figures 2, 3 and 4 has been made available online to allow for replication.

Randomization

This was not an experimental, but a descriptive study. Sequences were clustered using phylogenetic inference and SNP thresholds as described in the Methods. In one case where a sublineage inferred by rPinecone was clearly divided by geographical, temporal and genotypic variables, a manual approach was used to cluster sequences descended from common phylogenetic nodes (code provided online). Geospatial admixture between sublineages demonstrates that localised sampling bias is not substantial, as well as demonstrating the minimal impact of

	including samples from patients with CNS involvement
Blinding	This was a hypothesis generating analysis in which we had no prior knowledge of phylogenetic lineage or sublineage of samples used. The associated metadata was only linked to sequencing data after sequencing, variant inference, phylogenetic analysis and clustering had been performed.

Reporting for specific materials, systems and methods

We require information from authors about some types of materials, experimental systems and methods used in many studies. Here, indicate whether each material, system or method listed is relevant to your study. If you are not sure if a list item applies to your research, read the appropriate section before selecting a response.

Materials & experimental systems

n/a	Involved in the study
x	Antibodies
x	Eukaryotic cell lines
x	Palaeontology
	x Animals and other organisms
	x Human research participants
x	Clinical data

Methods

n/a	Involved in the study
x	ChIP-seq
x	Flow cytometry
x	MRI-based neuroimaging

Animals and other organisms

Policy information about studies involving animals; ARRIVE guidelines recommended for reporting animal research

Laboratory animals	Animals were not used directly for this study (US samples were collected and passaged in rabbits for prior studies and residual samples were subsequently sequenced for this one). In those studies, Male New Zealand white rabbits (approx 3 kg) were used. Animal care was provided in full accordance with established guidelines, and experimental procedures were conducted under protocols approved in advance by the University of Washington Institutional Animal Care and Use Committee.
Wild animals	Not applicable
Field-collected samples	Not applicable
Ethics oversight	Animal care was provided in full accordance with established guidelines, and experimental procedures were conducted under protocols approved by the University of Washington Institutional Animal Care and Use Committee.

Note that full information on the approval of the study protocol must also be provided in the manuscript.

Human research participants

Policy information about studies involving human research participants

Population characteristics	This was opportunistic sampling of people presenting with syphilis, or of residual DNA from diagnostic PCRs. Samples from Seattle were from a study of Syphilis patients with central nervous system disorders; we sequenced a mixture of Seattle samples from 21 patients with CNS involvement and 38 patients without.
Recruitment	Samples with low pathogen (treponema) load or low sequencing coverage were excluded due to the limitations of the sequencing technology - it is possible that low pathogen load samples might demonstrate different population characteristics (e.g. lineage specific effects). Samples from Seattle were from a study of Syphilis patients with central nervous system disorders; we sequenced a mixture of Seattle samples from 21 patients with CNS involvement and 38 patients without, and saw no observable difference in sequence data or phylogenetic clustering between the groups.
Ethics oversight	Use of the UK samples was approved by the NHS Research Ethics Committee (IRAS Project ID 195816). Use of US samples from Seattle had ethical approval at the University of Washington (UW IRB # STUDY00003216).

Note that full information on the approval of the study protocol must also be provided in the manuscript.
