# Supplementary material for: Genomic epidemiology of syphilis reveals independent emergence of macrolide resistance across multiple circulating lineages
Source: Nat Commun. 2019 Jul 22;10:3255. doi: 10.1038/s41467-019-11216-7 (PMC6646400; doi:10.1038/s41467-019-11216-7)
Supplement: Supplementary file 4 — Description of Additional Supplementary Files [file 41467_2019_11216_MOESM4_ESM.pdf]

Description of Additional Supplementary Files

File Name: Supplementary Data 1
Description: Full sample metadata (Excel Sheet) for this study, including sequence naming used in this paper, in other publications, and on GenBank, ENA Accessions for all new genomes, as well as results of lineage and sub-lineage typing and inference of genotypic macrolide resistance.

File Name: Supplementary Data 2
Description: List of genomic regions behaving in a non-clocklike manner and masked due to hypervariable, recombining or repetitive elements.
